# Supplementary material for: A first constraint on basal melt-water production of the Greenland ice sheet
Source: Nat Commun. 2021 Jun 8;12:3461. doi: 10.1038/s41467-021-23739-z (PMC8187594; doi:10.1038/s41467-021-23739-z)
Supplement: Supplementary file 1 — Supplementary Material [file 41467_2021_23739_MOESM1_ESM.pdf]

Supplementary materials for

"A First Constraint on Basal Melt-water Production of the Greenland Ice Sheet"

By Nanna B. Karlsson, Anne M. Solgaard, Kenneth D. Mankoff, Fabien Gillet-Chaulet, Joseph A. MacGregor, Jason E. Box, Michele Citterio, William T. Colgan, Signe H. Larsen, Kristian K. Kjeldsen, Niels J. Korsgaard, Douglas I. Benn, Ian Hewitt, and Robert S. Fausto.

The supplementary materials contain:

Supplementary Note 1 2
Supplementary Note 2 3
Supplementary Note 3 4
Supplementary References 5

Supplementary Note 1

Friction heat from Elmer/Ice and the shallow-ice approximation

In the comparison below, we have downscaled the surface and bed topographies, and the surface velocity dataset from Elmer/Ice to a 10km grid. The downscaled datasets have been used as input in the shallow-ice model. Thus, the difference between the two model outputs should mainly be due to the difference in model resolution and stress regime.

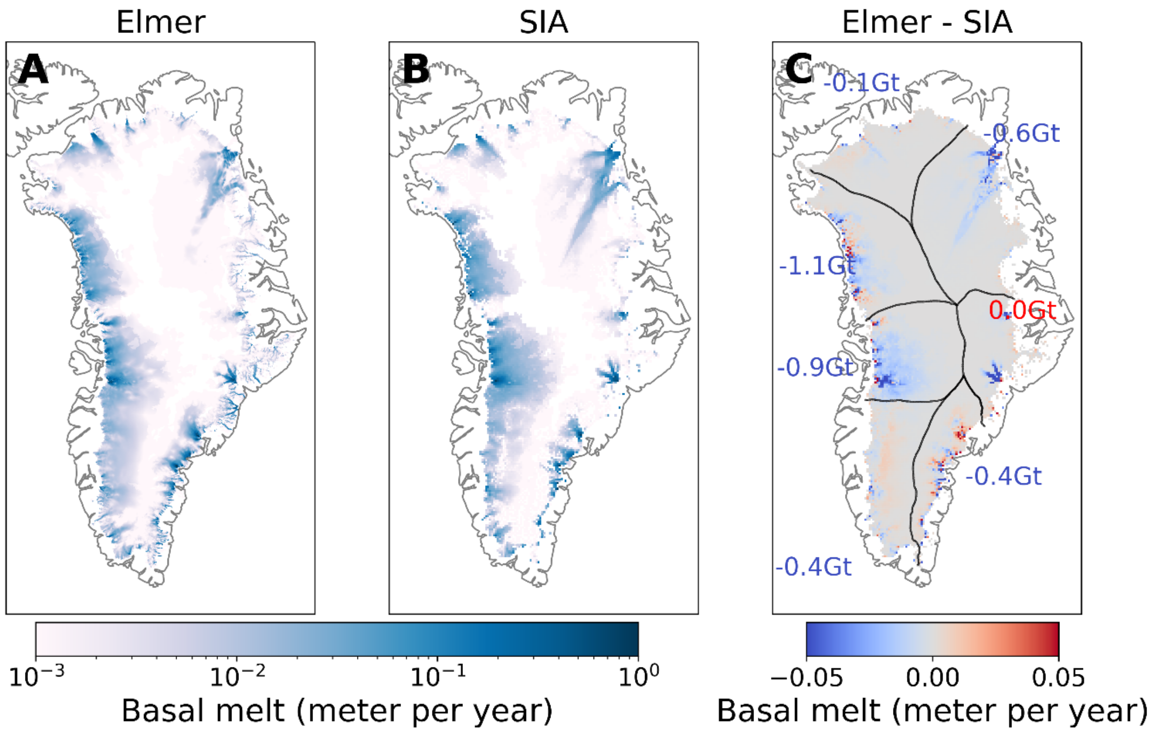

Supplementary Figure 1: Basal melt rates from (A) Elmer/Ice and from (B) the shallow-ice approximation. (C) shows the difference between the two. Numbers indicate difference in basal melt discharge for each sector in Gt (red indicates that Elmer/Ice estimates are higher than shallow-ice estimates).

Supplementary Table 1: Basal discharge for each sector derived from Elmer/Ice model and the shallow-ice approximation.

**Sector**	**Elmer/Ice (Gt per year)**	**Shallow-ice (Gt per year)**	**Difference (Gt per year)**
CE	1.2	1.2	0.0
CW	2.4	3.3	-0.9
NE	1.0	1.6	-0.6
NO	0.6	0.8	-0.1
NW	2.1	3.1	-1.1
SE	2.2	2.6	-0.4
SW	1.3	1.7	-0.4
**Total**	**10.9**	**14.3**	**-3.4**

Supplementary Note 2

Uncertainty estimates for geothermal flux

Below, we show the three different scenarios for the geothermal flux. The scenarios have been constructed based on the range of possible geothermal flux values as well as likely basal conditions.

Supplementary Table 2: Basal melt discharge from geothermal flux based on the mean, cold and warm scenarios. The "% change" column compares with the mean state.

Sector	Mean (Gt per year)	Cold (Gt per year)	% change	Warm (Gt per year)	% change
CE	0.5	0.2	-65 %	1.0	93 %
CW	0.7	0.5	-32 %	1.0	38 %
NE	1.3	0.8	-38 %	1.9	47 %
NO	0.4	0.2	-57 %	0.7	69 %
NW	0.6	0.4	-37 %	0.8	43 %
SE	0.7	0.3	-52 %	1.1	69 %
SW	1.2	0.8	-31 %	1.6	37 %
**Total**	**5.3**	**3.1**		**8.1**	

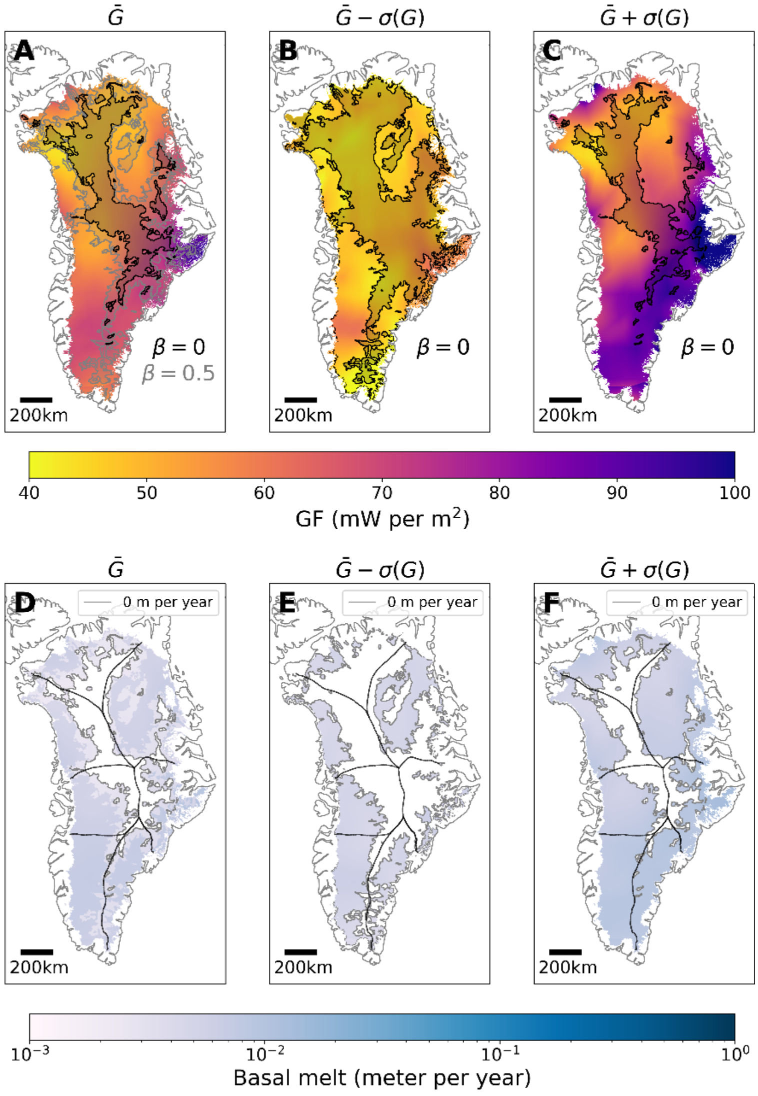

Supplementary Figure 2: Range of possible geothermal flux values with different basal conditions, representing mean (A), cold (B) and warm (C) scenarios.(D)-(F) Show corresponding basal melt rates on a logarithmic scale. β refers to Eq. 1 in the main text.

Supplementary Note 3

Englacial temperatures

The englacial temperatures that inform the choice of *A(T)* in shallow-ice approximation are derived from radar attenuation rates and published by MacGregor et al., (2015) [1]. The scattered data points (Supplementary Figure 3A) are linearly interpolated and extrapolated and smoothed by a 10km by 10km window (Supplementary Figure 3B).

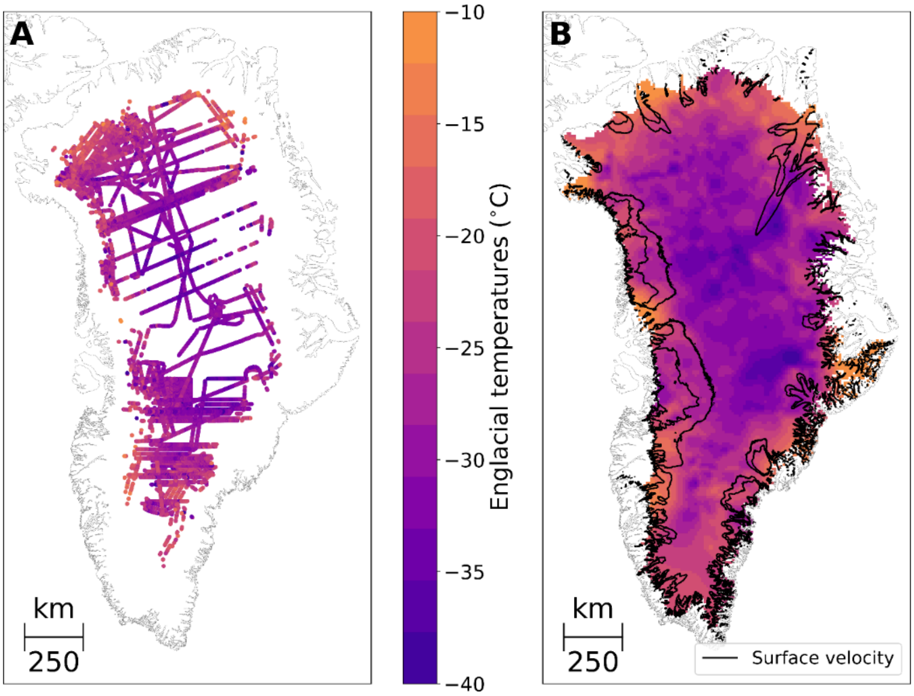

Supplementary Figure 3: (A) Englacial temperatures derived by MacGregor et al. (2015) from radar attenuation rates. (B) The regridded and smoothed englacial temperatures.

Constant offsets of 15°C, 20°C and 25°C are added to the regridded temperature product (Supplementary Figure 4). We use the likely basal conditions from MacGregor et al. (2016) [2] to guide our choice of offset. Choosing the lower boundary (15°C) would imply unrealistically cold ice over large parts of the ice sheet. The upper boundary (25°C) on the other hand would give warm ice in areas where the basal conditions should be below the pressure melting point. We therefore chose an offset of 20°C as the middle ground. We note that the resulting temperature (Supplementary Figure 4, middle) is neither the basal temperature nor the temperature of the entire ice column. It represents the temperature of the part of the ice that deforms the most, typically the lower 20% of the ice column.

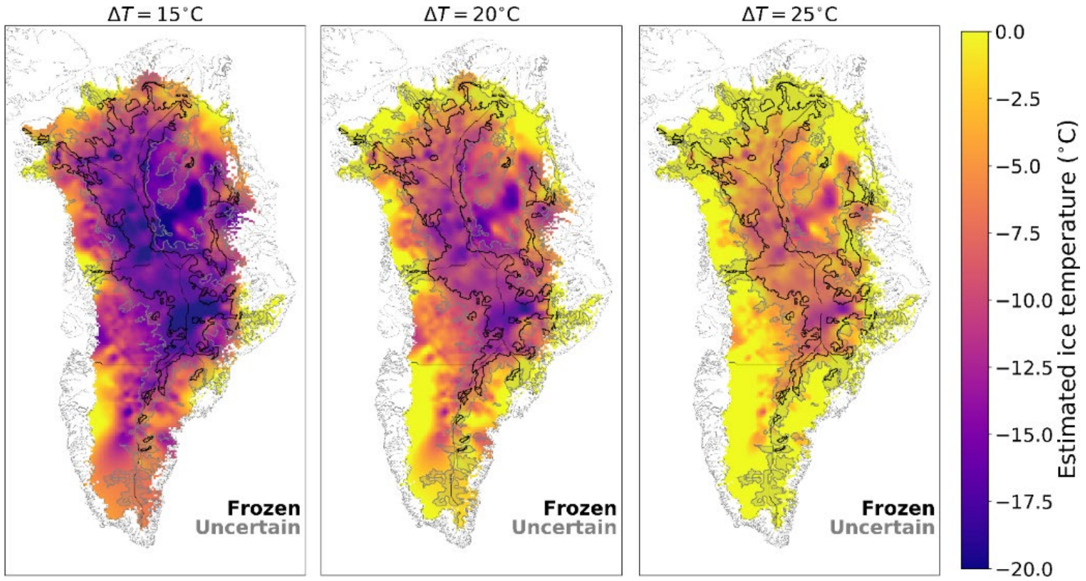

Supplementary Figure 4: Estimated temperature of the lower part of the ice column using offsets of 15°C, 20°C and 25°C. The black and grey contours indicate likely basal conditions also from radar observations as published by MacGregor et al. (2016).

Supplementary References

[1] MacGregor, J. A., et al. Radar attenuation and temperature within the Greenland ice sheet. Journal of Geophysical Research: Earth Surface 120, 983–1008. (2015).

[2] MacGregor, J. A., et al. A synthesis of the basal thermal state of the Greenland Ice Sheet. Journal of Geophysical Research: Earth Surface 121, 1328–1350 (2016).
